# Beyond cytokine blockade: could CAR-Tregs open a new era of tissue-targeted immune tolerance in psoriatic arthritis?

**DOI:** 10.3389/fimmu.2026.1772369

**Published:** 2026-02-11

**Authors:** Rubén Queiro, Sara Alonso, Mercedes Alperi

**Affiliations:** 1 Rheumatology Division, Hospital Universitario Central de Asturias, Oviedo, Spain; 2 Department of Medicine, Faculty of Medicine and Health Sciences, Oviedo University, Oviedo, Spain; 3 Translational Immunology Division, Health Research Institute of the Principality of Asturias (ISPA), Oviedo, Spain

**Keywords:** CAR-T cell therapy, CAR-Tregs, precision medicine, psoriatic arthritis, tissue-resident memory T cells

## 1 Introduction

Over the past two decades, psoriatic arthritis (PsA) therapy has evolved from broad immunosuppression to highly targeted biologic and small-molecule interventions. Agents blocking TNF, IL-17A/F, IL-23, IL-12/23, or JAK signaling have transformed outcomes for many patients. Yet these therapies share a key limitation: they suppress inflammation without restoring immune tolerance. Furthermore, treatment must be continued indefinitely, relapses are common, and a subset of patients progress to difficult-to-treat (D2T) disease despite multiple mechanisms of action (1).

Meanwhile, the field of engineered T-cell therapies, most notably chimeric antigen receptor (CAR) T-cell therapy, has expanded beyond oncology. Early successes of CD19-directed CAR-T cells in refractory lupus and other autoimmune diseases have renewed interest in cellular therapies that can recalibrate autoreactive networks rather than merely dampen downstream cytokine pathways (2). A related and rapidly emerging technology, CAR-engineered regulatory T cells (CAR-Tregs), offers a fundamentally different approach: localized, antigen-targeted induction of immune tolerance (3).

Recent advances in spatial immunology, single-cell transcriptomics, and immunopeptidomics have substantially refined our understanding of tissue-imprinted immune responses in chronic inflammatory diseases. In parallel, rapid progress in regulatory T-cell engineering, including optimized CAR designs and safety switches, has expanded the feasibility of tolerance-inducing cellular therapies beyond transplantation and oncology. Given the tissue-specific immunopathology of PsA, an important question arises as to whether PsA could become a future candidate for CAR-Treg–based interventions.

## 2 Psoriatic arthritis as a T-cell-centered disease: the immunologic rationale

Advances in single-cell transcriptomics and T-cell receptor (TCR) sequencing have demonstrated that PsA shows prominent clonal expansions of CD8^+^ T cells in synovial tissue and the enthesis (4–6). These cells display Tc17 and Tc1 effector phenotypes and produce IL-17A, IL-22, GM-CSF, and IFN-γ, reflecting activation by cytokines, stromal cues and biomechanical stress (4–6). The enthesis, once considered a purely structural organ, is now understood as an immune microenvironment enriched with IL-23R^+^ resident T cells responsive to microdamage and mechanical loading (7).

### 2.1 Tissue-resident memory T cells: a persistent immunologic layer

A key addition to this paradigm is the major role of tissue-resident memory T cells (TRM) in psoriatic disease. TRM are long-lived, non-circulating T-cell populations that persist in skin, enthesis, and synovium even after clinical resolution of inflammation. They typically express CD69, CD103, CCR6, and IL-23R, and are enriched for IL-17A/IL-22 effector programs (8).

Recent studies demonstrate that TRM in PsA remain chronically embedded within the tissue microenvironment; are clonally expanded, suggesting antigen-driven selection; act as a reservoir that can rapidly reactivate upon cytokine bursts or mechanical triggers; and contribute to the recurrence of inflammation in identical anatomical locations (8).

Importantly, current biologics—especially IL-23 and IL-17 inhibitors—can modulate TRM function, but they do not eliminate or reprogram TRM populations (8, 9). This provides a mechanistic explanation for phenomena such as tissue-specific relapses despite systemic remission.

Beyond clonal T-cell persistence, PsA is increasingly understood as a disease of aberrant stromal–immune integration, particularly at mechanically stressed sites such as the enthesis. Fibroblast-like synoviocytes, entheseal stromal cells, and resident mesenchymal populations actively shape the inflammatory milieu through cytokine production, extracellular matrix remodeling, and mechanotransduction pathways. Mechanical stress and microdamage can amplify IL-23 and IL-6 signaling, lowering activation thresholds for tissue-resident T cells and innate lymphoid populations, even in the absence of classical antigenic stimulation. This tight coupling between biomechanical cues, innate activation, and adaptive immune memory helps explain the spatially fixed and recurrent nature of inflammation in PsA and reinforces the concept of the enthesis as an immune–mechanical interface rather than a passive target tissue (7, 8).

## 3 Implications for therapy

TRM cells illustrate a fundamental limit of cytokine-blocking strategies as biologics suppress effector pathways but do not remodel the tissue-imprinted immune architecture sustaining chronicity (1, 9). This insight provides a rationale for therapies capable of modulating microenvironments directly—such as CAR-Tregs—which could, in principle, suppress TRM-dominated inflammation at its origin.

### 3.1 Why classic CAR-T therapy is not suitable for PsA

Classic CAR-T (cytotoxic CAR-T) therapies are highly effective in settings where a single, stable surface antigen uniquely identifies pathogenic cells (e.g., CD19 in B-cell malignancies) (2). PsA does not fit this model for several reasons: (i) lack of a universal pathogenic antigen, in fact.

PsA is a polyantigenic condition; candidate autoantigens such as LL-37, ADAMTSL5, keratinocyte-derived neoepitopes, or citrullinated extracellular matrix proteins lack universality across patients (7); (ii) multicellular, multiclonal pathogenesis: the disease involves TRM, resident CD8^+^ T cells, innate lymphoid cells, fibroblast-like synoviocytes, and stromal inflammatory circuits (4–7). Eliminating a single T-cell clone would be therefore insufficient; (iii) safety concerns:

cytokine release syndrome, neurotoxicity, and prolonged immunosuppression make classic CAR-T unacceptable for a chronic, non-fatal disease where safer alternatives exist; (iv) logistical and economic infeasibility: current CAR-T manufacturing costs exceed €300,000–500,000 per patient, a barrier far beyond what is justifiable for PsA.

### 3.2 CAR-Treg therapy: a more plausible path toward tissue-targeted immune tolerance

CAR-Tregs represent a fundamentally different therapeutic concept. Instead of killing target cells, CAR-Tregs are designed to re-establish immune tolerance by acting directly within inflamed tissues. CAR-Tregs are engineered from FOXP3^+^ regulatory T cells; express a chimeric receptor that guides them to specific antigens or microenvironmental structures; exert bystander suppression, reducing effector T-cell activation (including TRM) locally; release regulatory cytokines (IL-10, TGF-β), modulate antigen-presenting cells, and reshape stromal-immune interactions; and finally, preserve systemic immune function, unlike global immunosuppression (3).

A central and unresolved challenge in translating CAR-based strategies to PsA is the absence of a clearly defined, disease-specific autoantigen. This limitation is not merely due to incomplete discovery efforts but likely reflects the intrinsic biology of PsA, which appears to be driven by polyclonal, tissue-restricted immune responses shaped by local stress signals, post-translational modifications, and context-dependent neoepitopes rather than a single dominant antigen (4, 7). Antigenic heterogeneity across tissues, disease stages, and patients further complicates target identification and represents a fundamental barrier to classical CAR design paradigms.

Importantly, the conceptual rationale for CAR-Tregs in PsA does not necessarily rely on the identification of a unique pathogenic autoantigen. Unlike cytotoxic CAR-T cells, CAR-Tregs may exert therapeutic effects through bystander suppression, modulation of antigen-presenting cells, and regulation of stromal–immune circuits once localized within inflamed tissues (3, 10). Targeting shared tissue-associated structures, stress-induced extracellular matrix components, or stromal cell–associated molecules could theoretically enable CAR-Tregs to access pathogenic niches and restore local immune homeostasis, even in the context of antigenic diversity. Nonetheless, these strategies remain speculative and will require substantial experimental validation.

Preclinical studies have shown promising results in solid organ transplantation (HLA-A2-directed CAR-Tregs), colitis and inflammatory bowel disease models, autoimmune encephalomyelitis, cardiac autoimmunity, and early models of inflammatory arthritis (10). Notably, several of these experimental and early translational applications have been pursued in diseases that, similar to PsA, lack a single dominant autoantigen. In such contexts, the therapeutic premise relies less on precise antigen elimination and more on reshaping local immune regulation within affected tissues. These experiences highlight both the potential and the limitations of Treg-based cellular therapies in complex immune-mediated diseases and underscore the need for cautious interpretation when extrapolating these strategies to PsA.

## 4 Why PsA is a particularly strong conceptual candidate for CAR-Tregs

PsA exhibits several characteristics that make it theoretically well-aligned with CAR-Treg strategies. A spatially defined, tissue-specific disease: the enthesis acts as a micro-organ where immune cells, stromal signals, and mechanical forces converge (7). Localized modulation is therefore attractive. TRM-dominated microenvironments: persistent TRM populations maintain inflammatory potential even when systemic inflammation is controlled—ideal for a therapy that acts at the tissue level (8). Shared antigens between skin and musculoskeletal tissues: some autoantigen candidates overlap between psoriasis lesions and PsA joints, improving the feasibility of tissue-targeted CAR constructs (4). Unmet needs in difficult-to-treat PsA: patients failing ≥2 biologic classes represent a small but significant unmet need group and may justify advanced cellular therapies (11). Potential antigenic targets: although speculative, conceivable targets for CAR-Tregs include extracellular matrix neoepitopes in inflamed entheses, stress-induced or mechanically generated peptides, shared motifs detected via immunopeptidomics, and stromal or fibroblast-associated molecules enriched in PsA (12).

### 4.1 Which patients might one day benefit?

If CAR-Tregs became feasible, the most plausible PsA subgroups would include true D2T PsA unresponsive to multiple mechanisms, aggressive early-onset PsA with rapid structural progression, enthesitis-dominant endotypes driven by TRM/Tc17 signatures, and/or patients in whom systemic immunosuppression is contraindicated. Importantly, CAR-Treg therapy would be a precision intervention, not a broad treatment for all PsA.

### 4.2 Challenges ahead: scientific, technical, and ethical

Despite being a promising technology – still in its infancy – CAR-Treg therapy faces several challenges in PsA. (i) Scientific barriers: an additional and critical concern relates to the stability and functional fidelity of engineered regulatory T cells in chronically inflamed environments. PsA tissues are enriched in IL-23, IL-6, and other pro-inflammatory cytokines known to challenge FOXP3 stability and promote phenotypic drift toward effector programs. Although recent advances in Treg engineering aim to enhance lineage stability, metabolic fitness, and resistance to inflammatory reprogramming, long-term maintenance of a suppressive phenotype within PsA-relevant microenvironments remains uncertain. This issue represents a major biological risk that must be addressed before clinical translation can be realistically considered. (ii) Technical barriers: autologous Treg isolation and expansion. Need for cost-effective, off-the-shelf Treg platforms. Implementation of safety switches. (iii) Ethical and regulatory considerations: justifying gene-engineered therapy in a non-lethal disease. Requirements for long-term safety monitoring. Alignment with ATMP (advanced therapy medicinal product) frameworks.

## 5 Discussion

Psoriatic arthritis exemplifies the paradox of modern immunology where, despite the availability of highly effective cytokine-targeted therapies, truly lasting remission remains difficult to achieve for many patients (1). Current treatments excel at extinguishing inflammatory activity but fall short of reshaping the underlying immunological architecture that sustains chronicity. The enthesis and synovium are not passive tissues but immunologically active microenvironments in which T cells, stromal elements, and mechanical stimuli converge. These sites serve as reservoirs of inflammation capable of reactivation even when systemic cytokine levels are well controlled, illustrating the limitations of a pharmacologic model rooted primarily in cytokine blockade (1).

A growing body of single-cell and spatial immunology research highlights the centrality of clonal CD8^+^ T-cell expansions and tissue-resident memory T cells (TRM) in PsA pathogenesis (4–7). TRM cells create lasting, tissue-specific immune memory that remains well after clinical remission. Their ability to respond rapidly to local cues—mechanical stress, IL-23 bursts, or stromal activation—likely contributes to the well-known phenomenon of site-specific disease recurrence (7, 8). Importantly, current biologics modulate TRM effector function but do not erase TRM populations or reverse their residency programs. This biological reality underscores why even deep responses to IL-23 or IL-17 inhibition may not equate to immune resolution at the tissue level (1, 9).

These insights explain, at least in part, why PsA displays a plateau of therapeutic response. Biologics are highly effective at interrupting inflammatory cascades but do not comprehensively address the local immune ecosystems that propagate and sustain the disease (7). The disparity between systemic suppression and tissue-level persistence underscores a therapeutic limitation: current interventions are insufficient to achieve tissue-specific immune tolerance.

Within this framework, emerging cellular immunotherapies—specifically CAR-Tregs—offer a conceptual shift. Unlike cytotoxic CAR-T cells, CAR-Tregs are engineered not to deplete pathogenic cells but to actively restore immune regulation within diseased tissues (3, 10). Their mechanism of action is multifaceted: precise homing to antigens or microenvironmental structures, bystander suppression of local effector T cells, modulation of antigen-presenting cells, remodeling of fibroblast–immune interactions, and secretion of regulatory cytokines such as IL-10 and TGF-β (3, 10). These effects are inherently tissue-targeted and therefore map closely onto the immunobiology of PsA.

Preclinical studies have shown that CAR-Tregs can promote antigen-specific tolerance in transplantation, autoimmune encephalomyelitis, colitis, and cardiac inflammation (10). Their safety profile appears more favorable than traditional CAR-T platforms, with a low risk of cytokine release syndrome and a reduced likelihood of off-target tissue damage (3, 10). Importantly, CAR-Tregs offer the potential to reshape the immune landscape of a particular tissue, not just to suppress its inflammatory outputs (3). This makes them uniquely suited for conditions driven by durable, tissue-embedded immune programs such as PsA.

PsA also presents several features that could position it as a future candidate for CAR-Treg development. It is a spatially defined disease, most prominently affecting the enthesis, where immune and mechanical cues intersect (7). Disease-driving T-cell populations, including TRM and Tc17/Tc1 subsets, are enriched and clonally expanded locally (4). Some antigenic pathways appear to be shared between skin and musculoskeletal tissues, offering a conceptual foothold for the design of tissue-homing CAR constructs. Finally, a subset of patients with difficult-to-treat PsA—those failing multiple biologic mechanisms or displaying aggressive structural progression—represent a clinically meaningful unmet need in whom advanced therapies could be justified (11).

However, many challenges remain. The absence of a universal PsA antigen complicates CAR targeting; antigen discovery efforts must advance substantially before rational CAR construction is feasible. FOXP3 stability, phenotypic drift, and the long-term persistence of engineered Tregs are active areas of investigation (13–15). Technical barriers in Treg expansion, manufacturing, and cost remain substantial, and ethical considerations will be central when applying genetically engineered therapies to a chronic, non-lethal disease. Regulatory pathways under the ATMP framework will also shape the translational trajectory.

Despite these limitations, scientific convergence is compelling. Advances in tissue-peptidomics, TCR clonotyping, and spatial immunology are rapidly expanding our understanding of antigenic landscapes within PsA tissues (4–7). Parallel progress in Treg engineering—such as optimized CAR designs, safety switches, and “universal donor” platforms—may soon reduce some of the technical obstacles (13–15). Together, these emerging capabilities suggest that PsA could become a model condition for exploring targeted immune-tolerance strategies.

In summary, CAR-Treg therapy is not imminent for PsA, and its feasibility remains speculative. Yet its underlying logic aligns uniquely with the immunobiology of the disease. If future research continues to elucidate tissue-specific antigenic drivers and refine Treg engineering technologies, CAR-Tregs may represent a new frontier in PsA management: one that moves beyond cytokine blockade toward the restoration of local immune homeostasis (**Table 1**). Whether PsA ultimately becomes a proving ground for this approach will depend on the next decade of discovery at the intersection of immunology, rheumatology, and cellular biotechnology.

**Table 1:** Key concepts supporting CAR-Treg therapy as a future strategy in psoriatic arthritis.

Concept	Summary
Persistent tissue immunopathology	PsA is driven by clonal CD8^+^ T cells and TRM populations that persist in enthesis and synovium even after systemic remission.
Limitations of cytokine blockade	Current biologics suppress effector pathways but do not reprogram tissue-resident immune circuits or restore immune tolerance.
Tissue specificity of PsA	The enthesis acts as an immunologically active micro-organ where mechanical stress, stromal signaling, and T-cell infiltration converge.
Rationale for CAR-Tregs	CAR-Tregs can localize to inflamed tissues, exert bystander suppression, modulate APCs, and release IL-10/TGF-β to promote local tolerance.
Advantages over classic CAR-T	CAR-Tregs avoid cytotoxicity, reduce risk of CRS, and target immune regulation rather than cell depletion; no universal autoantigen required.
Who might benefit?	Difficult-to-treat PsA; aggressive early structural disease; enthesitis-dominant TRM-driven phenotypes; patients unsuitable for systemic immunosuppression.
Key challenges	Lack of defined antigens; FOXP3 stability; homing specificity; manufacturability; ethical considerations in non-lethal disease.
Future potential	Advances in antigen discovery, spatial immunology, and Treg engineering may make CAR-Tregs feasible as tissue-targeted immunotherapies in PsA.

Conceptual framework summarizing the immunopathological rationale, therapeutic gaps, and translational considerations supporting CAR-engineered regulatory T cells (CAR-Tregs) as a potential future strategy in psoriatic arthritis. The table integrates current evidence on tissue-resident immune mechanisms, limitations of cytokine-targeted therapies, and the theoretical advantages and challenges of localized immune tolerance induction.

PsA, Psoriatic arthritis; CAR, Chimeric antigen receptor; CAR-Tregs, Chimeric antigen receptor–engineered regulatory T cells; TRM, Tissue-resident memory T cells; APCs, Antigen-presenting cells; IL-10, Interleukin-10; TGF-β, Transforming growth factor beta; CRS, Cytokine release syndrome; FOXP3, Forkhead box P3.
